# Heterogeneous Vancomycin-Intermediate *Staphylococcus aureus* Uses the VraSR Regulatory System to Modulate Autophagy for Increased Intracellular Survival in Macrophage-Like Cell Line RAW264.7

**DOI:** 10.3389/fmicb.2019.01222

**Published:** 2019-05-31

**Authors:** Yuanyuan Dai, Caihong Gao, Li Chen, Wenjiao Chang, Wenwei Yu, Xiaoling Ma, Jiabin Li

**Affiliations:** ^1^Department of Infectious Diseases, First Affiliated Hospital of Anhui Medical University, Hefei, China; ^2^Department of Clinical Laboratory, First Affiliated Hospital of University of Science and Technology of China, Hefei, China; ^3^Anhui Center for Surveillance of Bacterial Resistance, Hefei, China; ^4^Department of Infectious Diseases, Chaohu Hospital of Anhui Medical University, Hefei, China

**Keywords:** *Staphylococcus aureus*, autophagy, VraSR, regulation, macrophage

## Abstract

The VraSR two-component system is a vancomycin resistance-associated sensor/regulator that is upregulated in vancomycin-intermediate *Staphylococcus aureus* (VISA) and heterogeneous VISA (hVISA) strains. VISA/hVISA show reduced susceptibility to vancomycin and an increased ability to evade host immune responses, resulting in enhanced clinical persistence. However, the underlying mechanism remains unclear. Recent studies have reported that *S. aureus* strains have developed some strategies to survive within the host cell by using autophagy processes. In this study, we confirmed that clinical isolates with high *vraR* expression showed increased survival in murine macrophage-like RAW264.7 cells. We constructed isogenic *vraSR* deletion strain Mu3Δ*vraSR* and *vraSR*-complemented strain Mu3Δ*vraSR-*C to ascertain whether *S. aureus* uses the VraSR system to modulate autophagy for increasing intracellular survival in RAW264.7. Overall, the survival of Mu3ΔvraSR in RAW264.7 cells was reduced at all infection time points compared with that of the Mu3 wild-type strain. Mu3Δ*vraSR*-infected RAW264.7 cells also showed decreased transcription of autophagy-related genes *Becn1* and *Atg5*, decreased LC3-II turnover and increased p62 degradation, and fewer visible punctate LC3 structures. In addition, we found that inhibition of autophagic flux significantly increased the survival of Mu3Δ*vraSR* in RAW264.7 cells. Together, these results demonstrate that *S. aureus* uses the VraSR system to modulate host-cell autophagy processes for increasing its own survival within macrophages. Our study provides novel insights into the impact of VraSR on bacterial infection and will help to further elucidate the relationship between bacteria and the host immune response. Moreover, understanding the autophagic pathway in *vraSR* associated immunity has potentially important implications for preventing or treating VISA/hVISA infection.

## Introduction

*Staphylococcus aureus* is an important human pathogen responsible for both hospital-associated and community-acquired infections (Monaco et al., 2017). *S. aureus* causes a wide range of diseases, from minor skin and soft tissue infections to severe systemic illnesses such as pneumonia, arthritis, endocarditis, and bacteremia (Lowy, 1998). In recent years, indiscriminate and extensive use of vancomycin has resulted in the emergence and development of vancomycin-intermediate *S. aureus* (VISA) and heterogeneous VISA (hVISA) strains, many of which show an increased ability to evade host immune surveillance and enhanced clinical persistence (Gardete et al., 2012; Cameron et al., 2017; Katayama et al., 2017).

Autophagy is a basic physiological process in eukaryotes and plays an important role in cellular repair and homeostasis (Ohsumi, 2014). Degraded intracellular components are removed and recycled into newly emerging double-membrane vacuoles called autophagosomes (Mizushima et al., 2010). These autophagosomes mature to fuse with lysosomes and are digested. This process of autophagosome formation and eventual degradation is termed autophagic flux (Mizushima et al., 2010). Studies have demonstrated that many pathogens have evolved strategies to harness autophagic processes for survival inside the host cell (Campoy and Colombo, 2009; Mostowy, 2013; Gomes and Dikic, 2014; Mostowy, 2014; Soong et al., 2015; Siqueira et al., 2018). In particular, Neumann et al. and Schnaith et al. reported that *S. aureus* can take advantage of the autophagic mechanism to aid in its own replication or intracellular survival (Schnaith et al., 2007; Neumann et al., 2016).

The two-component regulatory system VraSR is a vancomycin resistance-associated sensor (VraS)/regulator (VraR) that is highly expressed in VISA/hVISA strains. In this study, we found that clinical isolates with high *vraR* expression showed increased survival in murine macrophage-like RAW264.7 cells and deletion of *vraSR* in *S. aureus* resulted in decreased survival in RAW264.7, indicating that VraSR could enhance intracellular *S. aureus* survival. We hypothesize that the VraSR regulatory system could be involved in regulation of host autophagy pathways to promote the survival of *S. aureus*. To examine this hypothesis, the autophagic responses of RAW264.7 cells infected with hVISA reference strain Mu3, *vraSR* deletion mutant Mu3Δ*vraSR*, or *vraSR-*complemented strain Mu3Δ*vraSR-*C were investigated. The results showed that *S. aureus* uses the VraSR regulatory system to induce autophagy and inhibit autophagic flux, thereby increasing bacterial intracellular survival in RAW264.7. This finding provides novel insights into the impact of VraSR on bacterial infection and may help to further elucidate the relationship between bacteria and the host immune response.

## Materials and Methods

### Bacterial Strains, Plasmids, and Culture Conditions

The bacterial strains and plasmids used in this study are listed in Supplementary Table S1. All *S. aureus* strains were cultured with shaking (200 rpm) at 37°C in tryptic soy broth (TSB). *Escherichia coli* strains were cultured with shaking (200 rpm) in Luria-Bertani medium at 37°C. The culture media were supplemented with appropriate antibiotics when required (ampicillin, 100 μg/l; chloramphenicol, 10 μg/l; and anhydrotetracycline, 1 μg/ml).

### Construction of the *vraSR* Mutant Strain

The *vraSR* deletion mutant strain was constructed as described previously (Li et al., 2017). Briefly, the upstream and downstream fragments of *vraSR* were amplified from *S. aureus* Mu3 genomic DNA using the *vraSR*-UF/*vraSR*-UR and *vraSR*-DF/*vraSR*-DR primer sets, respectively, and ligated by overlap extension polymerase chain reaction (PCR) to form an up-down fragment. The resulting fragment was recombined into the temperature-sensitive shuttle plasmid pKOR1 using Gateway^®^ BP ClonaseTM II Enzyme Mix (Thermo Fisher Scientific) to generate recombinant plasmid pKOR1-*vraSR*. pKOR1-*vraSR* was then transformed into *S. aureus* strain RN4220 by electroporation for modification and then transformed into *S. aureus* strain Mu3. The mutant strains that had allelic replacement were screened via high temperature and anhydrotetracycline-resistant and chloramphenicol-sensitive colonies and were further confirmed by PCR, quantitative reverse-transcriptase PCR (qRT-PCR) and sequencing. All primers used in this study are listed in Supplementary Table S2.

### Complementation of the *vraSR* Deletion Strain

To generate a complementation strain, *vraSR* and its promoter region were amplified and cloned into the shuttle plasmid pLI50, producing recombinant plasmid pLI50-*vraSR.* The recombinant plasmid was then transferred into *E. coli* DH5α and DC10B successively, and finally electroporated into the *S. aureus* Mu3Δ*vraSR* strain. Successful uptake of the complementation plasmid was confirmed by restriction mapping, PCR, and sequencing of PCR fragments. The presence of *vraSR* transcripts within the transformants was verified by qRT-PCR analysis.

### Growth Curve Analysis

*S. aureus* strains were incubated overnight in 5 ml of TSB at 37°C with shaking at 200 rpm. The overnight cultures were diluted 1/100 in 30 ml of fresh TSB and incubated at 37°C with shaking at 220-rpm. The optical densities (OD_600__nm_) of the *S. aureus* cultures were then monitored at 1 h intervals for a total of 18 h.

### Cell Culture

Murine macrophage-like cell line RAW264.7 was cultured in Dulbecco’s modified Eagle’s medium (DMEM high glucose; HyClone) supplemented with 10% (v/v) fetal calf serum (FCS; HyClone). Cells were cultured in a humidified incubator containing 5% CO_2_ at 37°C.

### Assessment of Bacterial Intracellular Survival

Intracellular killing assays were performed as described previously (Munzenmayer et al., 2016). For *S. aureus* infection, early stationary phase bacteria (OD_600__nm_ = 1.0–1.5) were harvested and washed once in cold phosphate-buffered saline (PBS). RAW264.7 cells were then infected with *S. aureus* at a multiplicity of infection (MOI) of 10. Following incubation for 1 h, infected cells were washed three times with PBS before the addition of 10% (v/v) FCS-DMEM supplemented with 10 μg/ml lysostaphin (Sigma-Aldrich) and 100 μg/ml gentamicin (Sigma-Aldrich) to each well. Plates were then incubated for 1 h to kill extracellular bacteria. Following incubation, the cells were washed with PBS and further incubated in fresh 10% (v/v) FCS-DMEM. At 0, 3, 6, 12, and 24 h post-infection (hpi), infected cells were washed three times with PBS to remove extracellular bacteria and dead cells and lysed by the addition of 0.5% (v/v) Triton X-100 (Sigma-Aldrich). The number of intracellular bacteria (expressed as colony-forming units, CFU) was determined by serial dilution and plating on TSB agar. In addition, replicate plates were incubated with 1.25 mM 3-methyladenine (3-MA, Sigma) or 100 nM bafilomycin A1 (Baf A1, Sigma) for 2 h prior to infection to block autophagy in the RAW264.7 cells.

### Transmission Electron Microscopy

RAW264.7 cells were incubated with the individual *S. aureus* strains at a MOI of 50 for 3 h before being collected by centrifugation and fixed with 2.5% glutaraldehyde in 0.1 M phosphate buffer. Cells were then post-fixed in 1% osmium tetroxide and dehydrated through a series of graded acetone washes. The dehydrated cells were embedded in epoxy resin, sectioned, and stained with uranyl acetate and lead citrate in preparation for observation under a transmission electron microscope (HT7700; Hitachi Co.). Autophagosomes were counted as described previously. (Yla-Anttila et al., 2009).

### RNA Extraction and qRT-PCR Assays

*S. aureus* RNA was extracted as described previously (Dai et al., 2017) to examine levels of *vraR* expression. RAW264.7 cells (2.5 × 10^6^ cells/well) in 6-well plates were infected with *S. aureus* at a MOI of 50 and incubated at 37°C with 5% CO_2_. At 0, 1.5, 3, 4.5, and 6 hpi, the culture medium was removed and RAW264.7 cells were washed twice with ice-cold PBS. Total RNA was extracted using Trizol reagent (Invitrogen) according to the manufacturer’s instructions, and RNA quantity and quality were measured using a NanoDrop ND-1000 spectrophotometer (PeqLab). RNA was reverse-transcribed into cDNA using PrimeScript Reverse Transcriptase as per the manufacturer’s instructions (TaKaRa).

qRT-PCR assays were performed using aliquots of cDNA and SYBR Premix EX TaqTM II (Takara) in an ABI 7500 qPCR instrument (Foster). Primers used for expression analysis are described in Supplementary Table S1. All gene expression was normalized against that of *Actb* (β-actin) and the 16S rRNA gene was used as an internal control. All qRT-PCR assays were repeated three times.

### Western Blot Analysis

RAW264.7 (2.5 × 10^6^ cells/well) were infected with *S. aureus* at a MOI of 50 in 6-well plates for 3 h and then collected and washed twice with ice-cold PBS. The total protein from the cells was extracted using a RIPA lysis buffer solution (Wuhan Boster), with the total protein concentration determined using a bicinchoninic acid protein assay kit (Thermo Fisher Scientific). Cell lysis solutions were separated using sodium dodecyl sulfate-polyacrylamide gel electrophoresis and transferred to poly-vinylidene difluoride membranes (Millipore). The membranes were blocked in 5% skim milk in tris-buffered saline Tween 20 buffer for 2 h at room temperature and then incubated overnight at 4°C with primary antibodies against LC3-I/II and p62/SQSTM1 (1:1000, Cell Signaling Technology). The membranes were then incubated with a 1:5000 dilution of horseradish peroxidase-conjugated secondary antibody for 1 h at room temperature. Densitometric analysis of the western blot was performed using Image Gauge software (Fujifilm). β-actin was used as an internal control, and the ratio of the intensity of the protein of interest to β-actin was calculated.

### mRFP-GFP-LC3 Puncta Formation Assays

RAW264.7 cells were plated in 6-well plates and allowed to reach 70% confluence by the time of transfection. The cells were then infected with mRFP-GFP-LC3 adenoviral vector (HanBio) at a MOI of 100 for 36 h, cultured with *S. aureus* (MOI = 50) for 3 h, and then fixed in 4% (w/v) polyoxymethylene for 30 min at 37°C. Images were captured on a Zeiss LSM 800 laser scanning confocal microscope (Carl Zeiss AG) using a 63× oil objective lens, followed by image analysis using Zeiss ZEN acquisition software. For quantification of LC3 punctate, mRFP-GFP-LC3 and mRFP-LC3 punctate dots were counted in 100 cells. Punctate dots were measured using Image J software (NIH).

### Statistical Analysis

All statistical analyses were performed using SPSS (Version 16.0; SPSS for Windows). When appropriate, one-way analysis of variance (ANOVA) and unpaired *t*-tests were analyzed to determine statistical significance, with a *P*-value cutoff of <0.05 to establish significance. Where appropriate, Bonferroni posttests were performed to directly compare experimental means.

## Results

### The VraSR Regulatory System Is Important for Bacterial Intracellular Survival and Proliferation

**FIGURE 1:**
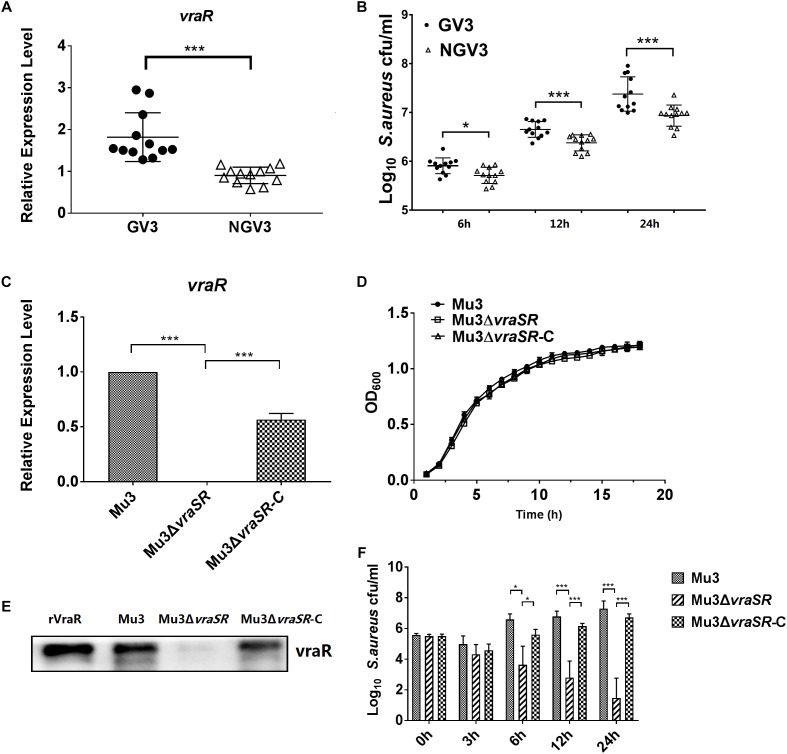
The VraSR regulatory system is important for intracellular survival and proliferation of *S. aureus*. **(A)** Comparison of the expression levels of *vraR* between the GV3 (12 isolates) and NGV3 (12 isolates) groups of clinical isolates by qRT-PCR assay. The data are the mean ± SD from three independent experiments (*n* = 3). Statistical significance was determined using a Student’s unpaired *t* test (*P* < 0.01). **(B)** Comparison of intracellular survival of *S. aureus* between GV3 (12 isolates) and NGV3 (12 isolates) clinical *S. aureus* isolates at each time point. The data are the mean ± SD from three independent experiments (*n* = 3). Statistical significance was determined using a Student’s unpaired *t-*test (*P* < 0.01). **(C)** Comparison of the expression levels of *vraR* in *S. aureus* strains Mu3, Mu3Δ*vraSR*, and Mu3Δ*vraSR*-C by qRT-PCR assay. The data are the mean ± SD from three independent experiments (*n* = 3). Statistical significance was determined by one-way ANOVA with Bonferroni posttest (*P* < 0.01). **(D)** Growth curve analysis of Mu3, Mu3Δ*vraSR*, and Mu3Δ*vraSR*-C. Bacteria were cultured in TSB at 37°C with shaking at 200 rpm. OD_600_ values were measured three times at each time point (*n* = 3). Statistical significance was determined by a one-way ANOVA with a Bonferroni posttest (*P* > 0.05). **(E)** Comparison of the levels of VraR expression in strains Mu3, Mu3Δ*vraSR*, and Mu3Δ*vraSR*-C by western blot. Gels are representative of three independent experiments (*n* = 3). **(F)** Survival of intracellular *S. aureus* were determined at each time point. The *X*-axis represents hours post-infection and the *Y*-axis represents log_10_ CFU/ml *S. aureus*. The data are presented as the mean ± SD. The SD was calculated from experiments performed in triplicate (*n* = 3). Statistical significance was determined by one-way ANOVA with Bonferroni posttest (*P* < 0.01). ^∗^*P* < 0.05; ^∗∗∗^*P* < 0.01.

In our previous study, a total of 24 clinical *S. aureus* isolates were screened on brain heart infusion agar containing 3 mg/l vancomycin (BHI-V3) (Chang et al., 2015). Based on the resulting data, we selected 12 isolates that grew on BHI-V3 (GV3) were selected as the test group, and other 12 isolates that did not grow on BHI-V3 (NGV3) for examination in the current study as the test and control groups, respectively. The relative ability of the two groups of clinical strains to persist intracellularly was quantified using a lysostaphin/gentamicin protection assay in RAW264.7 cells. We observed that the GV3 group showed increased survival rates in RAW264.7 cells at 24 hpi compared with the NGV3 group (Figure 1A,B). Therefore, to further investigate the impact of *vraR* expression on *S. aureus* intracellular growth, we constructed isogenic *vraSR* deletion strain Mu3Δ*vraSR* and *vraSR*-complemented strain Mu3Δ*vraSR-*C. We examined the mRNA expression and protein levels of VraR in the three *S. aureus* strains by q PCR analysis and western blotting to confirm the successful construction of the mutant strain (Figure 1C,D). To rule out the potential influence of the *vraSR* deletion on *S. aureus* growth rate, the growth of strains Mu3, Mu3Δ*vraSR*, and Mu3Δ*vraSR-*C was monitored hourly for 18 h. Overall, there were no substaintial differences in the growth of the three strains.

For *S. aureus* infection assay, we selected a low MOI (MOI = 10) instead of a high MOI (MOI = 50), because a high bacterial burden may cause earlier death of RAW264.7 cells, preventing intracellular replication of *S. aureus*. Our results showed that, in comparison with wild-type strain Mu3, fewer Mu3Δ*vraSR* cells were present within the infected RAW264.7 cells, and that the survival of Mu3Δ*vraSR* continued to decrease throughout the infection process. However, complemented mutant Mu3Δ*vraSR-*C showed similar survival rates to the wild-type strain Mu3 in RAW264.7 cells, indicating that VraSR is important for intracellular *S. aureus* survival and proliferation (Figure 1E).

### The VraSR Regulatory System Contributes to the Formation of Autophagic Vesicles in *S. aureus*-Infected Cells

**FIGURE 2:**
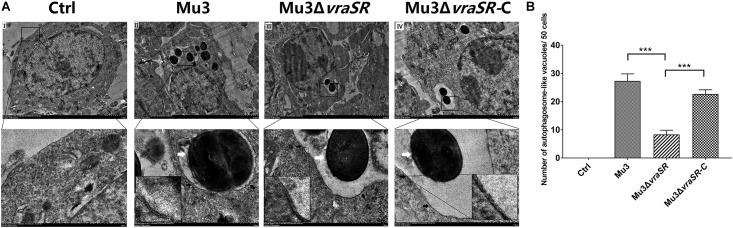
The VraSR regulatory system contributes to the formation of autophagosome-like vacuoles in *S. aureus*-infected cells. **(A)** Ultrastructural features were examined by transmission electron microscopy (TEM) under 3000× magnification. Uninfected **(I)** RAW264.7 cells were compared with RAW264.7 cells infected with Mu3 **(II)**, Mu3Δ*vraSR* **(III)**, or Mu3Δ*vraSR*-C **(IV)** for 3 h. Boxed areas from I, II, III, and IV are magnified from the respective main images (white arrow, intracellular *S. aureus*; black arrow, autophagosome-like vacuole). **(B)** Number of autophagosome-like vacuoles observed by TEM. The data are presented as the mean ± SD. The SD was calculated from experiments performed in triplicate (*n* = 3). Statistical significance was determined by a one-way ANOVA with Bonferroni posttest. ^∗∗∗^*P* < 0.01.

Ultrastructural features of infected cells were examined by transmission electron microscopy. Double-membraned structures, characteristic of autophagosomes, containing undigested *S. aureus* cells were observed within the RAW264.7 cells (Figure 2A). Quantification of *S. aureus*-containing autophagosome-like vacuoles per 50 infected cells showed that Mu3Δ*vraSR-*infected cells displayed significantly fewer *S. aureus*-containing autophagosome-like vacuoles compared with cells infected with Mu3 or Mu3Δ*vraSR-*C (*P* < 0.01), indicating that the VraSR regulatory system contributes to the formation of *S. aureus*-containing autophagosome-like vacuoles in *S. aureus*-infected cells (Figure 2B).

### The VraSR Regulatory System Contributes to the Expression of Autophagy-Related Genes

**FIGURE 3:**
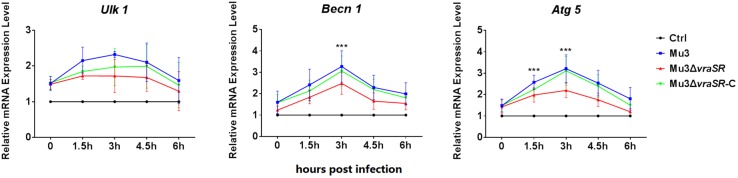
The VraSR regulatory system contributes to the expression of autophagy-related genes. qRT-PCR was performed to analyze the transcriptional levels of *Ulk1*, *Atg5*, and *Becn1* in Mu3-, Mu3Δ*vraSR*-, or Mu3Δ*vraSR*-C-infected cells at each time point. The data are presented as the mean ± SD. The SD was calculated from experiments performed five times (*n* = 5). Statistical significance was determined by one-way ANOVA with Bonferroni posttest. ^∗∗∗^*P* < 0.01.

As autophagy is a dynamic process, we performed a series of biochemical assays to examine the activation of autophagy at the molecular level (Levine et al., 2011). qRT-PCR assays were used to determine the transcriptional levels of autophagy-related genes *Ulk1*, *Becn1*, and *Atg5*. As shown in Figure 3, the transcriptional levels of the three genes were gradually upregulated between 0 and 3 hpi but declined after 4.5 hpi. Compared with Mu3-infected cells, the Mu3Δ*vraSR*-infected cells showed significantly lower transcriptional levels of *Becn1* and *Atg5* at 3 hpi, while gene expression in the Mu3Δ*vraSR-*C-infected cells was comparable with that in Mu3-infected cells, indicating that the VraSR regulatory system contributes to increase expression of autophagy-related genes.

### The VraSR Regulatory System Participates in Modulation of Autophagic Protein LC3 Turnover and p62 Degradation

**FIGURE 4:**
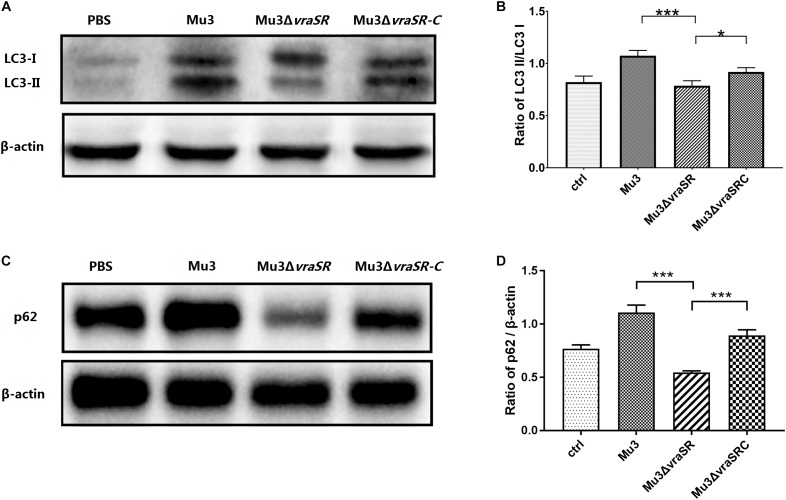
The VraSR regulatory system is involved in the regulation of autophagic protein LC3 turnover and P62 degradation. **(A)** Western blot analysis of the expression of LC3-I and LC3-II in RAW264.7 cells. Gels are representative of three independent experiments (*n* = 3). **(B)** Semi-quantitative analyses of LC3-II/LC3-I expression based on the density of bands on the western blot. The data are presented as the mean ± SD. The SD was calculated from experiments performed in triplicate (*n* = 3). Statistical significance was determined by one-way ANOVA with Bonferroni posttest. **(C)** Western blot analysis of the expression of p62 in RAW264.7 cells. β-actin was used as the loading control. Gels are representative of three independent experiments (*n* = 3). **(D)** Semi-quantitative analyses of p62/β-actin expression based on the density of bands on the western blot. The data are presented as the mean ± SD. The SD was calculated from experiments performed in triplicate (*n* = 3). Statistical significance was determined by one-way ANOVA with Bonferroni posttest. ^∗^*P* < 0.05; ^∗∗∗^*P* < 0.01.

LC3-II is a commonly used marker for autophagosome formation (Barth et al., 2010). Western blot analysis revealed that the levels of LC3-II in the Mu3Δ*vraSR*-infected cells were significantly lower than those in Mu3- and Mu3Δ*vraSR-*C-infected cells at 3 hpi (Figure 4A,B), indicating that the VraSR regulatory system is important for autophagic activity. Levels of other autophagic substrates that are degraded by autolysosomes, such as p62, can be used to monitor autophagic flux. We therefore monitored levels of p62 degradation by autolysosomes. At 3 hpi, the amount of p62 in Mu3Δ*vraSR*-infected cells was significantly lower than that in Mu3- and Mu3Δ*vraSR*C-infected cells (Figure 4C,D), indicating that the VraSR regulatory system may inhibit autophagic flux in RAW264.7.

### The VraSR Regulatory System Is Involved in Autophagic Flux Inhibition in *S. aureus* Infected Cells

**FIGURE 5:**
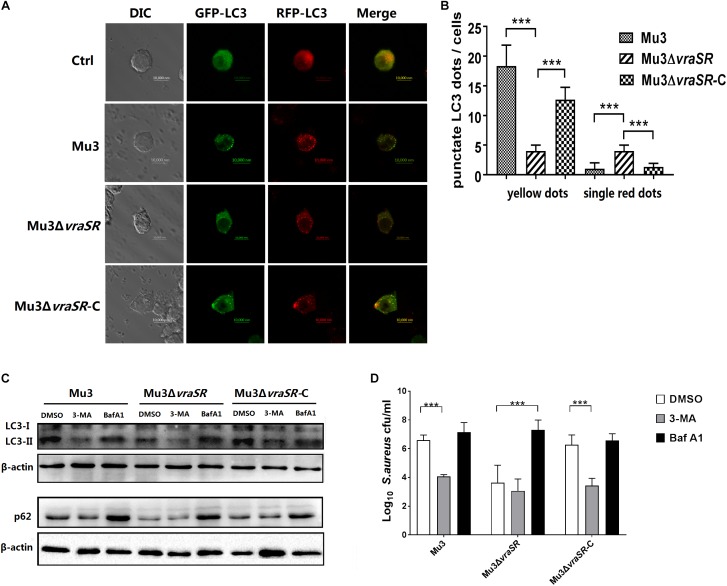
The VraSR regulatory system is involved in autophagic flux inhibition in *S. aureus*-infected cells. **(A)** RAW264.7 cells stably expressing mRFP-GFP-LC3 were infected with Mu3, Mu3*ΔvraSR*, or Mu3*ΔvraSR*-C for 3 h. Representative differential interference contrast (DIC) and corresponding GFP and RFP fluorescence images are shown. Bars, 10 μm. **(B)** Quantitative measurement of autophagic flux. The number of punctate dots was enumerated in at least 100 cells at 3 hpi. The *X*-axis represents the average number of puncta/cell. The data are presented as the mean ± SD. The SD was calculated from experiments performed in triplicate (*n* = 3). Statistical significance was determined by one-way ANOVA with Bonferroni posttest. **(C)** RAW264.7 cells were pretreated with 3-MA or Baf A1 for 2 h before being infected with *S. aureus* for 3 h. Cells were then lysed to examine LC3 and p62 protein levels by western blot. β-actin are used as the loading control. Gels were representative of three independent experiments (*n* = 3). **(D)** Quantification of intracellular bacteria in RAW264.7 cells infected with *S. aureus* for 6 h in the presence or absence of 3-MA or Baf A1. The *X*-axis represents different strains and the *Y*-axis represents log_10_ CFU/ml *S. aureus*. The data are presented as the mean ± SD. The SD was calculated from experiments performed in triplicate (*n* = 3). Statistical significance was determined by one-way ANOVA with Bonferroni posttest. ^∗∗∗^*P* < 0.01.

Autophagic flux was morphologically traced to distinguish between the two stages. mRFP-GFP-LC3 can be used as an indicator of autophagic flux because it appears yellow (mRFP and GFP signals merged) in autophagosomes and red (only mRFP signals) in autolysosomes, as a result of quenching of the GFP signal by the acidic pH of the lysosomes, while the RFP signal remains stable at an acidic pH. In this study, RAW264.7 cells stably expressing mRFP-GFP-LC3 were infected with different *S. aureus* strains. All autophagic structures could be measured by confocal microscope. Results showed that Mu3Δ*vraSR*-infected cells had fewer punctate LC3 structures (yellow dots) at 3 hpi compared with Mu3-infected cells, while the Mu3Δ*vraSR-*C-infected cells appeared similar to the Mu3-infected cells (Figure 5A,B), indicating that the VraSR regulatory system promotes the formation of autophagosomes. Moreover, we observed that Mu3Δ*vraSR-*infected cells had more single, red LC3 puncta compared with the other two groups, indicating that the VraSR regulatory system could block or delay the fusion of the autophagosome with the lysosome.

To further confirm the effect of the VraSR regulatory system on the autophagic pathway and to investigate whether the autophagic pathway can target intracellular *S. aureus*, RAW264.7 cells were pretreated with 3-MA (a well-established phosphatidylinositol 3-kinase (PI3K) inhibitor that prevents the induction of autophagy) or Baf A1 (a selective inhibitor of vacuolar H^+^-ATPase that prevents lysosomal acidification) for 2 h prior to infection with *S. aureus*. And we examined the effects of 3-MA and Baf A1 on cells viability by Cell Counting Kit-8 (CCK-8) assay. RAW264.7 cells viability was not affected at 12 h treatment with 3-MA or Baf A1 at concentrations up to 1.25 mM or 100 nM, respectively (Supplementary Figure S1). And the results of growth curve assay showed that 3-MA and Baf A1 had no effect on the growth of *S. aureus* (Supplementary Figure S2). We observed that 3-MA treatment decreased the levels of LC3-II (Figure 4C) and significantly decreased the intracellular survival of Mu3 and Mu3Δ*vraSR-*C in RAW264.7 cells (Figure 4D). In comparison, Baf A1 treatment significantly increased the accumulation of autophagic substrate p62 protein in all infected groups (Figure 4C), suggesting that autophagic flux was decreased in infected cells. We also determined that the intracellular survival of Mu3 and Mu3Δ*vraSR-*C was significantly decreased following pretreatment 3-MA while the survival of Mu3Δ*vraSR* inside cells pretreated with Baf A1 was significantly increased (Figure 4D). These results further demonstrated that the autophagy pathway of RAW264.7 cells is inhibited by 3-MA, which decreased the intracellular survival of *S. aureus*. Baf A1 inhibited autophagic flux and enhanced the survival of Mu3Δ*vraSR.* Taken together, these results indicate that *S. aureus* uses the VraSR regulatory system to block autophagic flux for increasing intracellular survival.

## Discussion

While autophagy is a cytosolic catabolic process in eukaryotic cells, it is also an innate defense mechanism against invading pathogenic bacteria (Gong et al., 2012). However, recent reports have shown that autophagy may play different roles during the infection of different bacterial pathogens, in addition to its known involvement in bacterial clearance, coordinating autonomous cell signaling, and, in some cases, promoting bacterial replication (Mostowy, 2013). Although autophagosomes are reported to be intracellular niches for *S. aureus*, the underlying mechanisms by which *S. aureus* triggers the autophagy machinery were poorly understood (Schnaith et al., 2007; Neumann et al., 2016). In the current study, we showed that *S. aureus* uses the VraSR regulatory system to enhance intracellular survival and increase the number of autophagic vesicles in *S. aureus*-infected cells, indicating that *S. aureus* uses the VraSR regulatory system to modulate autophagy in RAW264.7 cells.

The formation of the autophagosome involves the assembly of 36 autophagy-related (ATG) proteins into complexes that are essential for different steps of the process: the ULK1 complex triggers autophagy, the beclin 1 and class III phosphatidylinositol 3-OH kinase (PI3KC3) complex generates an essential lipid component of autophagosomes, and the ATG12-ATG5-ATG16L1 ubiquitin-like conjugation system mediates formation and elongation of the autophagosome. As autophagy is a dynamic process, we selected *Ulk1*, *Becn1*, and *Atg5* as markers of the three stages of autophagy, qRT-PCR analysis was then performed to examine the mRNA levels of each of the three genes. The transcript levels of *Becn1* and *Atg5* were significantly decreased in Mu3Δ*vraSR*-infected cells, indicating that the VraSR regulatory system contributes to increase the expression of autophagy-related genes. At the same time, we observed that the VraSR regulatory system participates in modulation of autophagic protein LC3 turnover and P62 degradation. Confocal microscopy-based morphological analyses revealed that the VraSR regulatory system plays an important role in *S. aureus*-induced autophagosome maturation and inhibition of autolysosome formation. Therefore, our findings suggest that the VraSR regulatory system positively contributes to bacterial survival in RAW264.7 cells in two ways: first, hVISA strains use the VraSR regulatory system to promote autophagy, thereby recruiting LC3 protein to develop the autophagosomes; and second, hVISA strains use the VraSR regulatory system to interfere with autophagosome and lysosome fusion, thereby enhancing the intracellular survival *S. aureus*.

According to the literature, various bacterial species exploit autophagy and promote the formation of autophagic vacuoles in which to multiply by regulating their cell wall components or virulence factors (Campoy and Colombo, 2009; Irving et al., 2014; Castrejon-Jimenez et al., 2015). For example, peptidoglycan is one of the most components of the *Listeria monocytogenes* cell wall. As such, peptidoglycan and its cleavage products are recognized by peptidoglycan-recognition protein, which induces autophagy (Yano et al., 2008). Intracellular *L. monocytogenes* then hijacks autophagy in macrophages by secreting the virulence factor listeriolysin O to evade killing (Zhang et al., 2019). In *S. aureus* strains, two-component system VraSR can up-regulate the synthesis of peptidoglycan, resulting in an increase in D-alanine-D-alanine residues (Kuroda et al., 2003). However, whether VraSR can induce autophagy via thickening of the peptidoglycan layer and increasing its cleavage products as well as *L. monocytogenes* needs further verification.

Our previous study demonstrated the capacity of VraSR to modulate *S. aureus* virulence by binding the P2–P3 intergenic region of the *agr* promoter (Dai et al., 2017). The Agr quorum-sensing system is a key regulatory system in Staphylococci, controlling the expression of a number of virulence factors. It is also essential for *S. aureus* survival within macrophages (Kubica et al., 2008). Some reports have also suggested that factors regulated by Agr are required for an autophagic response to *S. aureus* infection (Schnaith et al., 2007; O’Keeffe et al., 2015; Soong et al., 2015). Therefore, we speculate that the mechanism via which the VraSR regulatory system regulates autophagy may be related to the expression of Agr, which we plan to investigate in future.

In this work, we determined that *S. aureus* uses the VraSR regulatory system to block autophagic flux and delay the early stage of autophagosome formation, thereby promoting bacterial survival inside RAW264.7 cells. Although this study is limited by the use of only a single murine cell lineage and one *S. aureus* strain, to our knowledge, this is first report showing that the VraSR two-component system is responsible for the onset of autophagy in eukaryotic cells. Our findings provide new insights into the impact of VraSR on bacterial infection and will help to further elucidate the relationship between bacteria and host immune response. Importantly, our results suggest that VraSR may be a potential target for preventing or treating VISA/hVISA infection.

## Author Contributions

YD, XM, and JL contributed conception and design of the study. YD organized the database. CG and WC performed the statistical analysis. YD wrote the first draft of the manuscript. LC, WC, WY, and XM wrote sections of the manuscript. All authors contributed to manuscript revision, read, and approved the submitted version.

## Conflict of Interest Statement

The authors declare that the research was conducted in the absence of any commercial or financial relationships that could be construed as a potential conflict of interest.

## Supplementary Material

FIGURE S1 CCK8 analysis of cell viability of RAW264.7 cells treated with different concentrations of 3-MA **(A)** or Baf A1 **(B)** for 12 h. 3-MA did not show cytotoxicity at concentrations of up to 1.25 mM and Baf A1 did not show cytotoxicity at concentrations of up to 100 nM.

FIGURE S2 Growth curves of Mu3 **(A)**, Mu3ΔvraSR **(B)**, and Mu3ΔvraSR-C **(C)** cultured in presence or absence of 3-MA or Baf A1. Bacteria were grown in TSB at 37°C with shaking at 200 rpm. Values of OD_600__nm_ were measured every 1 h. Values are from three biological replicates ± SEM. Statistical significance was determined by a one-way ANOVA with Bonferroni posttest (*P* > 0.05).

TABLE S1 Strains an plasmids used in this study.

TABLE S2 Primers used in this study.
