# Supplementary figures and images for: Heterogeneous Vancomycin-Intermediate Staphylococcus aureus Uses the VraSR Regulatory System to Modulate Autophagy for Increased Intracellular Survival in Macrophage-Like Cell Line RAW264.7
Source: Front Microbiol. 2019 May 31;10:1222. doi: 10.3389/fmicb.2019.01222 (PMC6554704; doi:10.3389/fmicb.2019.01222)

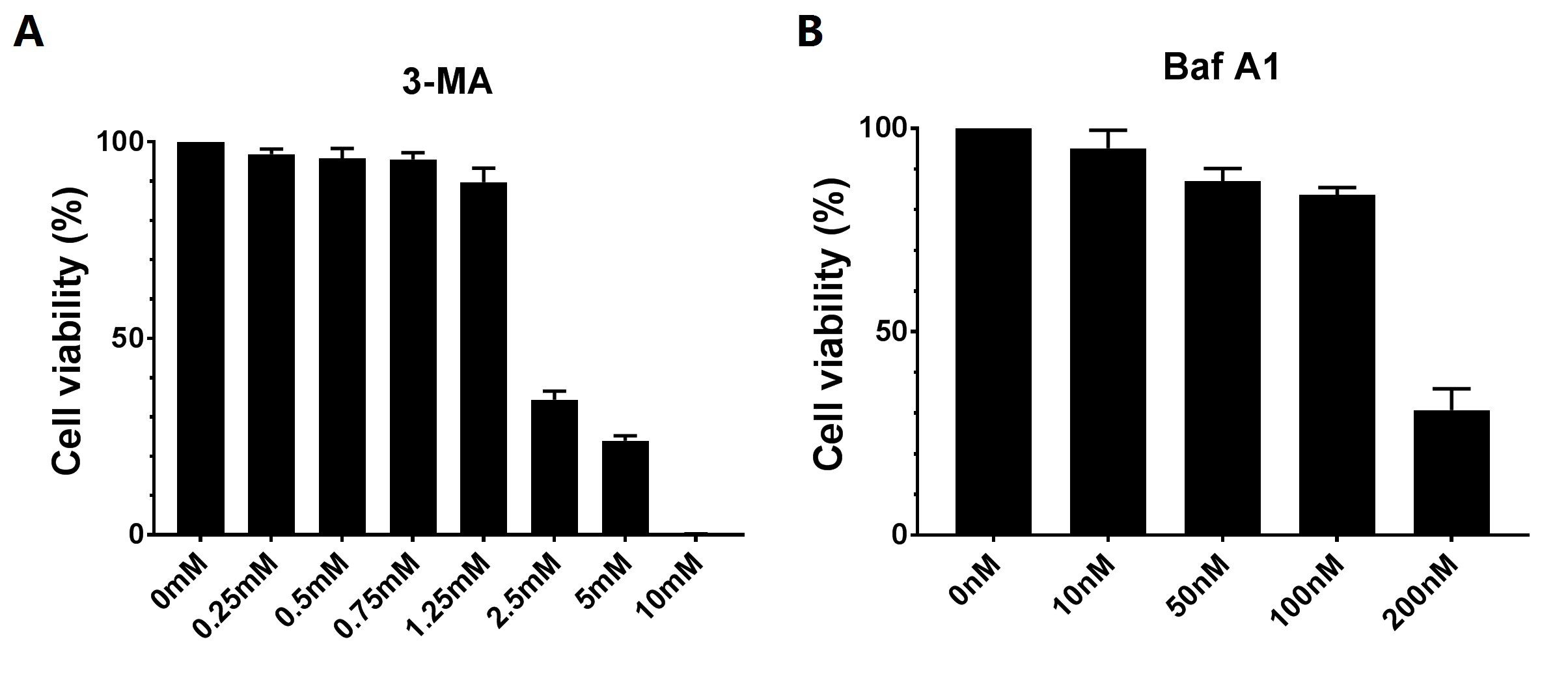

Supplement: FIGURE S1 — CCK8 analysis of cell viability of RAW264.7 cells treated with different concentrations of 3-MA (A) or Baf A1 (B) for 12 h. 3-MA did not show cytotoxicity at concentrations of up to 1.25 mM and Baf A1 did not show cytotoxicity at concentrations of up to 100 nM. [file Image_1.TIF]

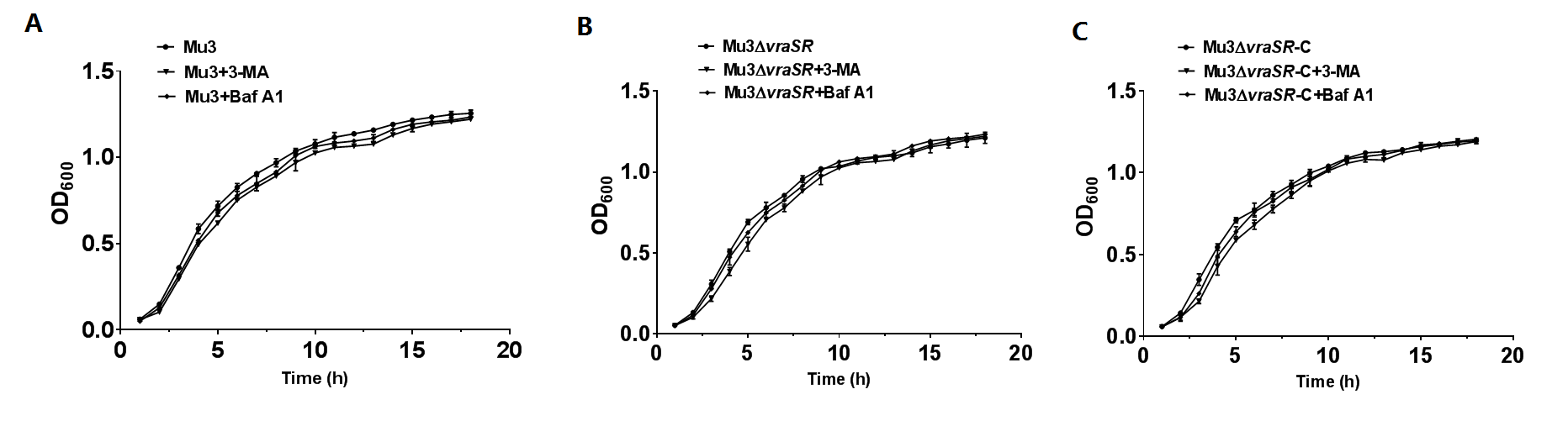

Supplement: FIGURE S2 — Growth curves of Mu3 (A), Mu3ΔvraSR (B), and Mu3ΔvraSR-C (C) cultured in presence or absence of 3-MA or Baf A1. Bacteria were grown in TSB at 37°C with shaking at 200 rpm. Values of OD600 nm were measured every 1 h. Values are from three biological replicates ± SEM. Statistical significance was determined by a one-way ANOVA with Bonferroni posttest (P > 0.05). [file Image_2.TIF]
