# Supplementary material for: Heterogeneous Vancomycin-Intermediate Staphylococcus aureus Uses the VraSR Regulatory System to Modulate Autophagy for Increased Intracellular Survival in Macrophage-Like Cell Line RAW264.7
Source: Front Microbiol. 2019 May 31;10:1222. doi: 10.3389/fmicb.2019.01222 (PMC6554704; doi:10.3389/fmicb.2019.01222)
Supplement: TABLE S1 — Strains an plasmids used in this study. [file Table_1.DOCX]

| **Strains and plasmid** | **Description** | **Source** |
| --- | --- | --- |
| Strains |  |  |
| *Escherichia coli* |  |  |
| DH5ɑ | Cloning strain | Laboratory stock |
| DC10B | dam+∆dcm−∆hsdRMS end A1 recA1; cloning strain | Laboratory stock |
| *Staphylococcus aureus* |  |  |
| 24 Clinical MRSA strains | Wild type, clinical MRSA strain isolated from clinical specimens | Laboratory stock |
| RN4220 | 8325-4, r^-^ | Laboratory stock |
| Mu3 | Wild type, ATCC700968 | Laboratory stock |
| Mu3ΔvraSR | Isogenic vraSR-deleted mutant in Mu3 | This study |
| Mu3ΔvraSR-C | *vraSR* mutant complementary with pKOR-*vraSR* | This study |
| Plasmids |  |  |
| pKOR1 | shuttle cloning vector, temp sensitive, Ampr Cmr | Laboratory stock |
| pKOR1-vraSR | pKOR1 containsing fragments 994-bp upstream and 1181-bp downstream of vraSR gene, for vraSR mutagenesis,Ampr Cmr | This study |
| pLI50 | shuttle cloning vector, Ampr Cmr | Laboratory stock |
| pLI50-vraSR | pLI50 with vraSR and its promoter, Ampr Cmr | This study |

**Table S1. Strains an plasmids used in this study**
