# Supplementary material for: Heterogeneous Vancomycin-Intermediate Staphylococcus aureus Uses the VraSR Regulatory System to Modulate Autophagy for Increased Intracellular Survival in Macrophage-Like Cell Line RAW264.7
Source: Front Microbiol. 2019 May 31;10:1222. doi: 10.3389/fmicb.2019.01222 (PMC6554704; doi:10.3389/fmicb.2019.01222)
Supplement: TABLE S2 — Primers used in this study. [file Table_2.DOC]

Table S2. Primers used in this study

| Name | Primer sequence(5'-3') | Application |
| --- | --- | --- |
| *vraSR*-UF | GGGGACAAGTTTGTACAAAAAAGCAGGCT**GCACCCGCTGAAACATCTAC** | *vraSR* deletion |
| *vraSR*-UR | cgatgaaccactacaatagaac**AGAACACAAGCTGTCATCTATGC** | *vraSR* deletion |
| *vraSR*-DF | gcatagatgacagcttgtgttct**GTTCTATTGTAGTGGTTCATCG** | *vraSR* deletion |
| *vraSR*-DR | GGGGACCACTTTGTACAAGAAAGCTGGGT**GACATCAACGAAGATACATAGC** | *vraSR* deletion |
| *vraSR-*C-F | GCGgaattcAGGTAAAGTGCAGGTTATATTGCCGGTTAA | Complementation |
| *vraSR-*C-R | GCGggtaccCTATTGAATTAAATTATGTTGGAATGCATAGATGA | Complementation |
| *vraR-F* | AAGACTAAACACCAACAAAACAGAG | qRT-PCR |
| *vraR-R* | GAAAAGTTACTTACGCCAATCACA | qRT-PCR |
| *16sRNA-F* | CGTGCTACAATGGACAATACAAA | qRT-PCR |
| *16sRNA-R* | ATCTACGATTACTAGCGATTCCA | qRT-PCR |
| *Ulk 1-F* | GCCCTTGATGAGATGTTCCA | qRT-PCR |
| *Ulk 1-R* | AATGTCTGCCTGGTCCGTGA | qRT-PCR |
| *Atg5-F* | AGAAAAAAGACCACAAGCAGC | qRT-PCR |
| *Atg5-R* | CTCCGTCGTGGTCTGATATATT | qRT-PCR |
| *Becn 1-F* | TACCGACTTGTTCCCTATGG | qRT-PCR |
| *Becn 1-R* | GGTCAAACTTGTTGTCCCAG | qRT-PCR |
| *β-actin-F* | AGCCTTCCTTCTTGGGTATGG | qRT-PCR |
| *β-actin-R* | CCTGTCAGCAATGCCTGGGTA | qRT-PCR |
